# Barriers and facilitators of improved nutritional support for patients newly diagnosed with cancer: a pre-implementation study

**DOI:** 10.1186/s12913-024-11288-2

**Published:** 2024-07-15

**Authors:** Benedicte Beichmann, Christine Henriksen, Ingvild Paur, Mari Mohn Paulsen

**Affiliations:** 1 Department of Nutrition, Institute of Basic Medical Sciences, University of Oslo, P.O. Box 1110, Blindern, Oslo, 0317 Norway; 2 Norwegian Advisory Unit on Disease-Related Undernutrition, Oslo, Norway; 3 Section for Clinical Nutrition, Department of Clinical Services, Division of Cancer Medicine, Oslo University Hospital, Oslo, Norway; 4 Department of Food Safety, Norwegian Institute of Public Health, Oslo, Norway; 5 Centre for Sustainable Diets, Norwegian Institute of Public Health, Oslo, Norway

**Keywords:** Malnutrition, Cancer, Qualitative study, CFIR, RCT

## Abstract

**Background:**

Disease-related malnutrition affects a significant number of patients with cancer and poses a major social problem worldwide. Despite both global and national guidelines to prevent and treat malnutrition, the prevalence is high, ranging from 20 to 70% in all patients with cancer. This study aimed to explore the current practice of nutritional support for patients with cancer at a large university hospital in Norway and to explore potential barriers and facilitators of the intervention in the Green Approach to Improved Nutritional support for patients with cancer (GAIN), prior to implementation in a clinical setting.

**Methods:**

The study used individual interviews and a focus group discussion to collect data. Study participants included different healthcare professionals and patients with cancer treated at a nutrition outpatient clinic. The Consolidated Framework for Implementation Research (CFIR) was used to guide the thematic data analysis.

**Results:**

Barriers connected to the current nutritional support were limited resources and undefined roles concerning responsibility for providing nutritional support among healthcare professionals. Facilitators included a desire for change regarding the current nutritional practice. The GAIN intervention was perceived as feasible for patients and healthcare professionals. Potential barriers included limited knowledge of technology, lack of motivation among patients, and a potential added burden experienced by the participating patients.

**Conclusions:**

The identification of the potential barriers and facilitators of the current nutritional support to patients with cancer will be used to plan the implementation of improved nutritional support in a randomized controlled trial for patients with cancer prior to clinical implementation. The current findings may be of value to others trying to implement either or both nutritional support and digital application tools in a clinical healthcare setting.

**Trial registration:**

The study was registered in the National Institutes of Health Clinical trials 08/09/22. The identification code is NCT05544318.

**Supplementary Information:**

The online version contains supplementary material available at 10.1186/s12913-024-11288-2.

## Background

One of many burdens patients with cancer may face during their course of disease is disease-related malnutrition [1, 2]. Disease-related malnutrition, from here on referred to as malnutrition, occurs when there is an unbalance between the energy need and the intake or uptake of energy, leading to unfavorable alterations in body composition and - functions [3]. The causes of this unbalanced condition vary; however, several aspects contribute to the risk of becoming malnourished. Cancer diagnosis, cancer stage, the patient’s age, inflammation, and cancer treatment are some of the potential factors [1]. Independent of the causes, malnutrition increases the risk of morbidities and worsens cancer prognosis, and thus, decreases the risk of overall survival [4–6].

Globally, malnutrition is estimated to affect 20–70% of all patients with cancer [1]. The large range is closely related to treatment setting, tumor type, and stage of disease as demonstrated by Mashall et al [7]. Their findings indicated that 31% of patients with cancer were malnourished in an Australian population. Within this population, individuals with breast cancer exhibited the lowest prevalence of 13%, while patients with upper gastrointestinal cancer had the highest prevalence at 62% [7].

Further, malnutrition is a major social problem, with significant costs for societies worldwide. In the United Kingdom, expenditure on malnutrition in 2012 amounted to approximately 22.1 billion euros, which represented 15% of the total costs used on health and social care [8]. Similarly, a recent report found that malnutrition costs the Norwegian society around 2.9 billion euros per year, of which health services costs related to malnutrition (i.e. costs connected to tertiary healthcare, nursing homes, and home care) were estimated to reach 1.2 billion euros in 2022 [9]. The major driver for these costs is prolonged hospital stays for malnourished patients [9].

In accordance with the latest guidelines on nutrition for patients with cancer from the European Society for Clinical Nutrition and Metabolism (ESPEN) [10], all hospitalized or ambulatory patients should be screened for malnutrition. If a risk of malnutrition has been identified, a nutritional assessment should be carried out. Thereafter, an individual plan to ensure sufficient nutrition should be outlined in cooperation with the patient and their caretakers according to Norwegian guidelines [11].

Despite international and national guidelines to prevent and treat malnutrition, patients at risk of malnutrition continue to be under-recognized, and thus, malnutrition is undertreated. Research from both Scandinavia and the United States shows that screening for malnutrition is not practiced routinely [12–14]. Simultaneously, studies show that only 41–53% of the identified at-risk patients in Norwegian hospitals receive nutritional treatment [15, 16] even though research indicates beneficial effects from intensified nutritional counseling during cancer treatment [17, 18]. The lack of systematic screening and treatment of malnutrition might be one of many reasons for the high prevalence of malnutrition across the healthcare system.

Green Approach to Improved Nutritional support (GAIN) is a randomized clinical trial that will test the effects of an improved nutritional support, simultaneously with the clinical cancer treatment, for patients with newly diagnosed colon, rectal, anal, and cervical cancer. The improved nutritional support will involve continuous dialogue with a registered dietitian from the time of diagnosis and throughout the next 6 months of cancer treatment. Nutritional support will be given through both digital systems and physical attendance.

Implementing nutritional interventions into clinical practice can be demanding [19, 20]. To increase the potential for successful implementation of the intervention, barriers and facilitators should be investigated at different levels, i.e. level of the patient, the individual healthcare professional and the healthcare team [21, 22].

This study aimed to explore the current practice of nutritional support for patients with cancer at a large university hospital in Norway, and further, to explore potential barriers and facilitators of implementing an improved nutritional support for patients newly diagnosed with cancer throughout the clinical cancer pathway to obtain a better understanding of how to implement the clinical trial, GAIN.

## Methods

### Study design and participants

This study reports qualitative findings from individual interviews and a focus group discussion regarding patients´ and healthcare professionals´ views on the current nutritional support for patients with cancer and potential barriers and facilitators of the intervention in the GAIN study. Study participants included nurses, physicians, registered dietitians, and a health secretary, in addition to patients with cancer treated at a nutrition outpatient clinic at a large university hospital in Norway. The participants were purposively selected, as they represented a selection of stakeholders that are involved in the clinical cancer pathway. Healthcare professionals were identified and suggested by their leader on request from the GAIN project, and thereafter asked to participate by e-mail. Patients were asked to participate by their treating dietitian on behalf of the GAIN project. Some of the healthcare professionals from this study will be involved in future investigations in the ongoing clinical GAIN study, whereas the included patients will not.

**Fig. 1:**
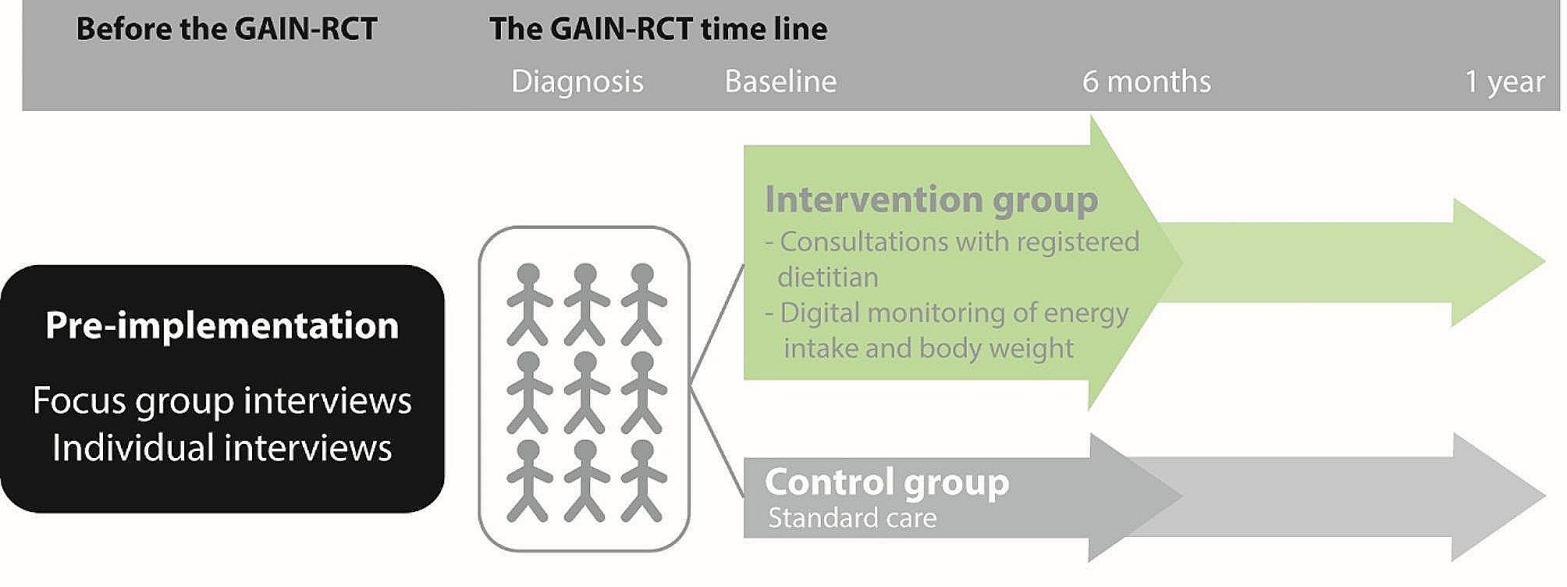
Timeline and overview of the GAIN project. *Abbreviations* GAIN: Green Approach to Improved Nutritional support, RCT: Randomized Clinical Trial

This study was an important part of the preparations, development and implementation of GAIN, a randomized clinical trial aiming to reduce malnutrition among patients with cancer. The intervention in GAIN includes an improved nutritional support, through early and frequent communication with a registered dietitian during cancer therapy, both digitally and physically. The physical attendances include a baseline visit close to the time of diagnosis and an obligatory visit approximately 6 months from baseline. Twelve months after baseline the participants will receive questionnaires concerning quality of life, physical, psychological and social functions. Frequent dietary assessment through a digital dietary tool, will take place every 25th day from baseline through the next 6 months or as often as the participants are in need for or desire. If the assessment reveals insufficient intake of energy, protein, or fluids compared to the estimated needs, the participant will be offered an additional visit to a registered dietitian. Participants in the control group will attend 2 visits: one at baseline and 6 months after baseline. The control group will receive standard care (i.e., nutritional screening for malnutrition and support if indicated), according to the clinical cancer pathway, and thereby no nutrition support specifically from GAIN. The clinical trial in the GAIN project adheres to the CONSORT guidelines [23]. The timeline of the GAIN project is illustrated in Fig. 1.

### MyFood

**Fig. 2:**
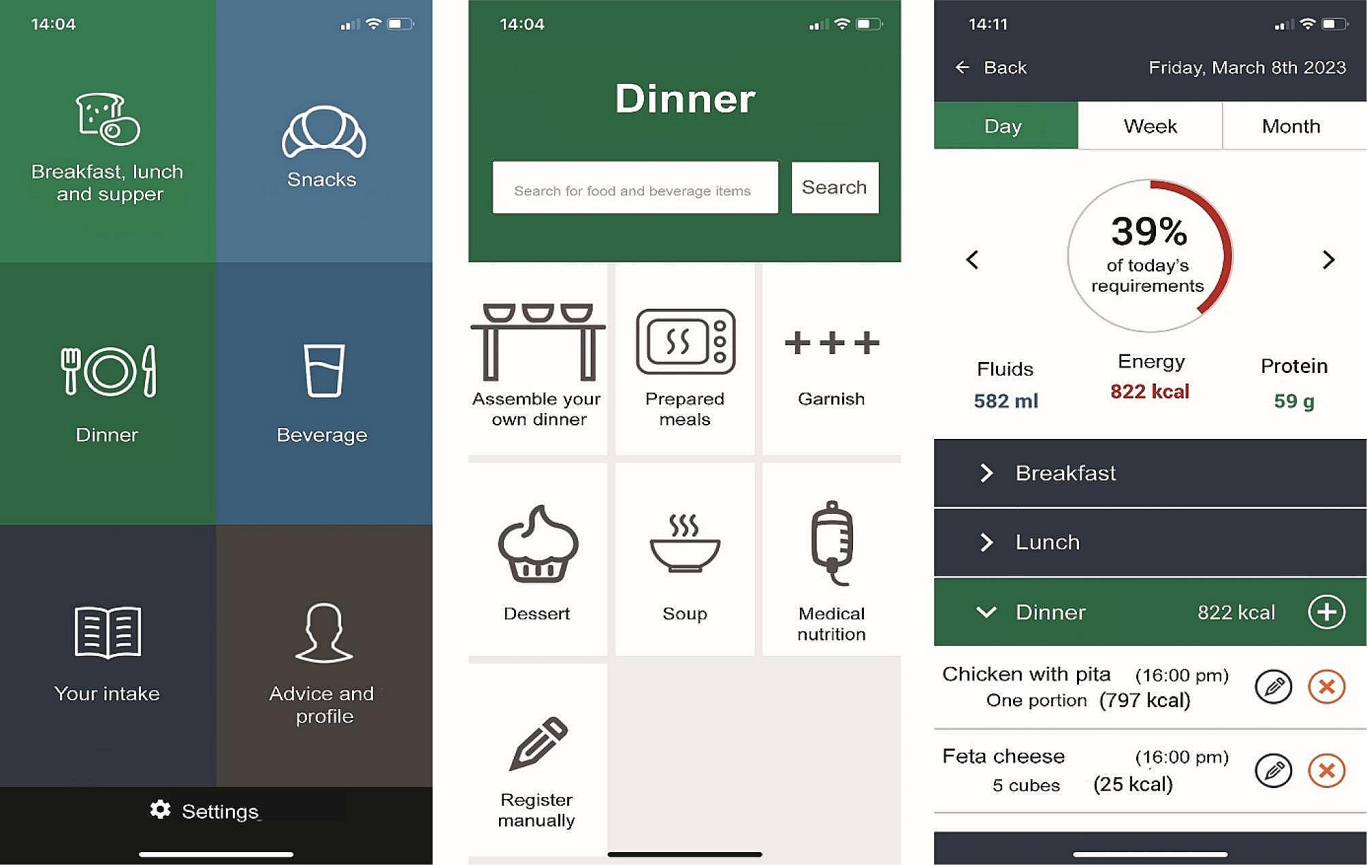
The MyFood tool from a patient view. From the left [1] Main menu of functions; [2] Menu for dinner recording; [3] Evaluation of recorded intake compared to estimated requirements for fluids, energy, and protein

MyFood is a digital tool designed to prevent and treat malnutrition [24, 25] that will be used in the intervention group in GAIN. This is accomplished through the assessment and evaluation of dietary intake, either reported by the patient, by their treating healthcare professional, or their next-of-kin. MyFood includes both an application that reports the recorded intake, and a website that allows healthcare professionals to evaluate and monitor the reports [24]. Screenshots of MyFood are illustrated in Fig. 2. The application is available for use at home, where the patient can compose their homemade meals, or at the hospital with the possibility to choose from the different hospital menus included in MyFood. Allergies, specific diets, and/or symptoms may also be registered [24].

### Interview guides and procedure

Healthcare professionals were either interviewed individually or included in a focus group. Prior to the interviews, the healthcare professionals were asked to fill out a form (supplementary file 1) including questions about work experience, occupation, age, and gender. The interview guides were based on the Consolidated Framework for Implementation Research (CFIR) and developed for this study and can be found in the supplementary material (supplementary file 2–4). In brief, the interview guide for healthcare professionals included questions about the respective hospital department, with a focus on the nutritional support routines including the use of digital tools, i.e., video calls and applications. A question about the perceived impression of satisfaction with nutritional support among patients was also included. After a short demonstration of MyFood, respondents were asked about their perceptions of using the digital dietary tool. At last, they were encouraged to share thoughts about the GAIN intervention, with emphasis on barriers and facilitators.

All patients were interviewed individually. For patients, the interview guide included questions about previous experience with organized nutritional support. Background information regarding diagnosis, age, and gender was received from their treating dietitian. Patients were asked to describe their nutritional treatment so far and share thoughts about the use of digital tools in nutritional support and their opinions regarding digital versus physical consultations. The patients received an individual demonstration of MyFood and were asked to elaborate on the use and design.

The first author (BB) performed the focus group interview, with help from the last author (MMP) taking notes. During the focus group, participants were offered hot drinks (i.e., tea or coffee) and a snack. The interviews took place in a meeting room (focus group) or consultation room (individual interviews). The first author also performed the individual patient interviews, in addition to one individual interview with a healthcare professional. The remaining six of the seven individual interviews with the healthcare professionals were conducted by the last author.

All interviews were recorded with a digital voice recorder (Olympus VN-741PC or WS-852). A Dictaphone application, developed by the University Center for Information Technology at the University of Oslo, was used as a backup. The focus group interview lasted an hour, whereas the individual interviews lasted from 16 to 45 min. The first author transcribed all the recordings verbatim, using the software f4transkript, version 7, 2018 (Marburg).

### The consolidated framework for implementation research

The CFIR is a framework to guide systematical assessments of potential barriers and facilitators before implementing strategies for an upcoming intervention [26]. The framework provides a compilation of 39 constructs, organized within five domains. The five domains are (1) intervention characteristics, (2) outer setting, (3) inner setting, (4) characteristics of the individuals involved, and (5) process of implementation [19].

### Data analysis

To analyze the transcripts, thematic analysis as described by Braun and Clarke [27], was used. Initially, each transcript was thoroughly read through. To perform the data analysis, NVivo version 12 (QSR International) was used. Based on CFIR version 2009 [19], each of the 5 domains, with the 39 belonging constructs were generated into the software program. Thereafter, data were coded into one of the following themes: Intervention characteristics, inner setting, outer setting, or process.

Trustworthiness in the analysis, including credibility, confirmability, dependability, and transferability was emphasized. This involved the inclusion of both healthcare professionals and patients in the interviews, audio taping, and transcribing the material verbatim. It also involved the first (BB), the second (CH), and the last (MMP) authors in the development of the interview guides. The data were analysed systematically, involving both the first (BB) and the last (MMP) authors in the analysis and the interpretation of the results.

### Ethics

The study was performed in accordance with the Helsinki Declaration and was approved by the Norwegian Regional Ethical Committee, reference identification: 267889. The GAIN study is registered in the National Institutes of Health Clinical trials, identifier: NCT05544318, and in the Norwegian Agency for Shared Services in Education and Research (SIKT), reference identification: 219582. All participants signed written consents.

## Results

### Participant characteristics

Nineteen participants contributed to the data material. This included 12 individual interviews with seven healthcare professionals and five patients, and one focus group with seven personnel (i.e., five registered dietitians, one nurse, and one health secretary) employed at a nutrition outpatient clinic. All participants were either employed or treated at a large university hospital in Norway. The patients that contributed to the interviews all had a cancer diagnosis and received support in accordance with their nutrition-related symptoms or diagnosis. Table 1 describes the characteristics of the participants.

**Table 1:** Characteristics of participants

Characteristics	Healthcare professionals	Healthcare professionals	Patients
	Individual interviews	Focus group	Individual interviews
	(*n* = 7)	(*n* = 7)	(*n* = 5)
Gender (n)			
* Male*	1	0	4
* Female*	6	7	1
Age, years			
*Median (range)*	37 (26–55)	32 (27–54)	61 (44–83)
Occupation (n)			
* Nurse*	5	1	-
* Physician*	2	0	-
* Registered dietitian*	0	5	-
* Health secretary*	0	1	-
Experience* (n)			
<5 years	3	6	-
≥5 years	4	1	-
Diagnosis (n)			
* Gynecologic cancer*	-	-	1
* Head and Neck cancer*	-	-	3
* Neuroendocrine cancer*	-	-	1

*from the present hospital ward

**Fig. 3:**
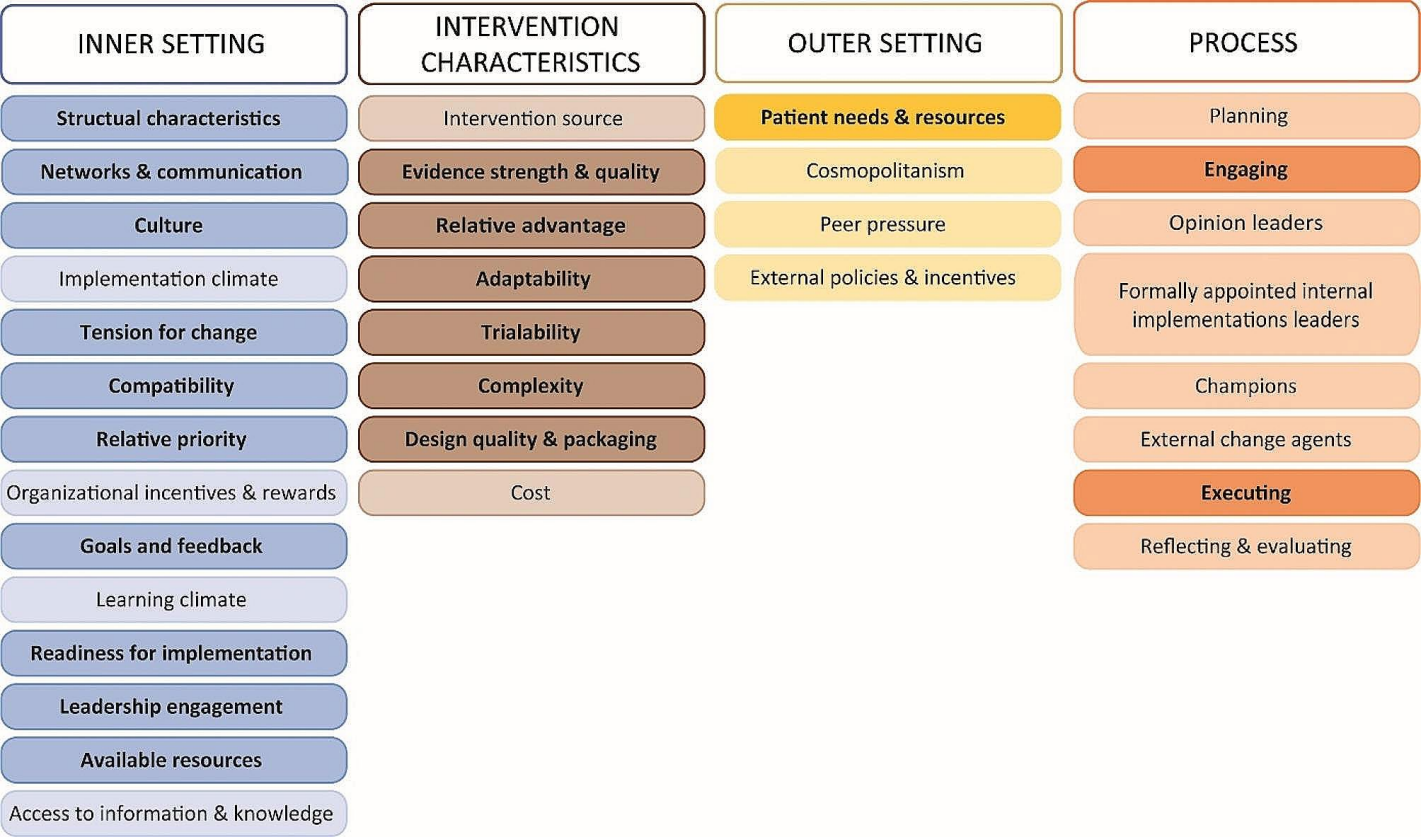
An overview of The Consolidated Framework for Implementation Research 2009-version [20], including four of its five domains. Data were sorted into 19 of the 39 consorts (illustrated in bold text with dark colors), the remaining consorts (light colors and regular text) were not applied

Figure 3 gives an overview of the applied CFIR domains and constructs. In total, 19 of the 39 constructs were identified in the data material and included in this study. No data were sorted into the theme “*Characteristics of individuals”*, it is therefore not included in the figure.

### Barriers and facilitators of providing the current nutritional support

To obtain an understanding of the present situation related to nutritional support in the relevant healthcare facilities (inner setting), barriers and facilitators of the current nutritional support within these settings were explored. This showed that most patients were screened for malnutrition by healthcare professionals as recommended by guidelines, however, several other aspects of nutritional support were noted as challenging. The CFIR describes available resources as the resources dedicated to the ongoing project, such as money, training, education, and time [19]. Healthcare professionals described limited time and work resources as specific barriers to providing nutritional support and care (Inner setting, CFIR construct available resources).

*It is probably a question of resources. The constant pressure to get these patients [patients with cancer] through the system and get them treated … I would like to wish for more dietitians*,* it is not always easy to get hold of them – although they try to be available* (physician 1).

A respondent stated that frequent follow-ups were important in order to detect possible side effects from treatment and offer help to relieve nutrition-impact symptoms (e.g. diarrhea, nausea, or reduced appetite). However, a lot of different information was given during the follow-ups, and little time was left to discuss and focus on nutrition (Inner setting, CFIR construct available resources).

*We don’t talk much about nutrition [with the patient] … When we have the first conversations about treatment plans*,* there is so much to say (physician 2).*

All healthcare professionals were asked if nutritional-related work and tasks were encouraged by their leaders and management. Most of the respondents claimed that there was little or no management involvement concerning nutrition (Inner setting, CFIR construct leadership engagement).

*… No*,* I don’t think nutrition is talked about that much [from leaders and management]* (nurse 1).

However, there were some differences across wards, as one of the respondents mentioned an increased focus from the hospital management over the last years.

The majority of respondents concurred that the responsibility for providing nutritional support to patients was shared between physicians and nurses. Moreover, if necessary, they referred the patient to a registered dietitian. However, there were some discrepancies between the different respondents regarding the social network and communication within the different wards. Some agreed that the nurses had an initial responsibility for nutritional support, as they often carry out nutritional screening, whereas others immediately indicated that the physician had the main responsibility (Inner setting, CFIR construct network, and communication).

*… The day-to-day follow-up is very much the nurses [main responsibility for nutrition]. We [nurses] follow the patient*,* we weigh them*,* and we make them responsible. But we also pass it [i.e.*,* clinically relevant information] on to the physicians. And we ask the physicians to refer to a dietitian*,* or we do the screenings… We are the ones paying attention to the patient* (nurse 2).

*It [nutritional support] is a collaborative project*,* but it has to be the physicians in a way*,* who must have the total responsibility… with a close collaboration or delegation to the dietitians* (physician 1).

The need for change regarding nutritional support became evident when one of the respondents explained how dietary recording often is challenging and time-consuming in a hospital setting. The challenges could be even more complex if the patient received meals from friends or family, or bought food outside the hospital (Inner setting, CFIR construct tension for change).

*Yes*,* I personally think there are challenges… the spouse brings homemade fish soup*,* and it’s a bit like - what is it really? [Ingredients in the soup]* ….

*They [the patients] have been to Kiwi [a local grocery store] and bought things - and then we [nurses] have to Google our way to what each food item contains. We spend a lot of time on that. A lot* (nurse 1).

### Barriers and facilitators of the improved nutritional support

To warrant a successful implementation of the improved nutritional support in the GAIN study, intervention characteristics were explored among the respondents. The digital dietary registration tool MyFood was one of these characteristics. The respondents described the potential advantages of digital assessment tools as ease of use, empowerment of the patient, efficiency, and transparency compared to traditional dietary recording on paper. This applied to both patients and healthcare professionals included in the study – and they perceived digital assessment tools to be relevant regardless of location (e.g. hospitalized or home-based) (Intervention characteristics, CFIR construct relative advantage).

*For me*,* it would have been nice to have an overview of what I eat … water intake*,* and weight too. I think it’s useful to pay attention. And if someone else can benefit from it [i.e. healthcare professionals]*,* that’s great* (patient 2).

*… it’s super useful if they [patients] manage to record*,* and we [registered dietitians] can just extract that information and not have to spend time on dietary recalls*,* but rather spend time on the results of the registration. Then it’s great* (registered dietitian 1).

The design of MyFood received only positive comments. It was perceived as simple, intuitive, and neat (Intervention characteristics, CFIR construct design quality, and packaging).

*It [the MyFood app] is nicely organized and user-friendly - and there are not too many choices. It’s manageable and efficient* (patient 1).

However, the use of digital communication in general, instead of physical consultations was pointed out as a potential barrier (intervention characteristics, CFIR construct design quality, and packaging).

*It works*,* but it’s not the same as being face-to-face… You lose the personal relationship with the therapist* (patient 2).

Concerns were also raised regarding the lack of technological skills and the use of digital tools among older patients (Intervention characteristics, CFIR construct complexity).

*The elderly will probably find it demanding. After all*,* there are a number of [research] studies that already gather information using apps – and they [the elderly] don’t want to participate* (nurse 3).

When the healthcare professionals were asked about their belief in the GAIN intervention, most respondents implied that it would be of great value to the patients – especially throughout the cancer treatment (Intervention characteristics, CFIR construct evidence strength and quality).

*I think it [nutrition] has a big impact on how they [patients with cancer] get through the treatment. If they don’t eat*,* they often feel nauseous*,* experience fatigue*,* become sedentary*,* then they get problems with their digestion … it kind of becomes a vicious circle then. So*,* I think that it [improved nutritional support] has a lot of impact* (nurse 3).

It was also mentioned that the improved nutritional support and frequent contact between the treating healthcare professionals and patients at home would lead to better systemic monitoring of the patient compared to present routines.

Despite positive feedback concerning the intervention in GAIN, several of the healthcare respondents expressed concern about the patient burden related to the different elements of the intervention (Outer setting, CFIR construct patient needs and resources).

*For patients treated with radiotherapy*,* I think it might be a bit too much … At the beginning of the treatment there is a lot to consider. I experience that they feel that they lack an overview. They come in for radiotherapy here every day. Then there are blood tests once a week. Then they get chemotherapy at another place once a week*,* and then there is a doctor’s appointment once a week. And all these appointments are at different places in the hospital* (nurse 3).

The reported concern applied to patients receiving frequent oncology treatment, but also encompassed the period between different treatment regimens (such as radiotherapy and chemotherapy), during which patients remain at home. Several potential barriers were reported, e.g., a reduction in the *joy of eating* due to strict dietary recording, and more stress as a result of several settings where food is brought up (for instance at home from family, oncology nurses, oncologists, dietitians and by the GAIN employees), and an increase in experienced fatigue attributed to their participation in the GAIN study. This concern also applied to the use of MyFood (Outer setting, CFIR construct patient needs and resources).

*I think for some patients it [MyFood] will be a fantastic tool and then I think there are some patients who don’t*,* who will find that it becomes too much*,* that it [the dietary recording] sort of takes over*,* that there is too much focus on nutrition. They [the patients] almost can’t bear to eat or can’t bear to eat a banana*,* because they can’t bear to record it afterward* (nurse 5).

Concerning the extra burden on the patient, it was also pointed out that the patients, who might benefit the most from the improved nutritional support, possibly could be the ones with the lowest compliance to the intervention.

To facilitate the implementation of the GAIN intervention, respondents mentioned the need for training and support (Process, CFIR construct Executing).

*Technical support [concerning digital software in general] to the users [both healthcare professionals and patients]. I think that is extremely important for them [the patients]. Just to log in and to download an app - it may stop there for some. For a group who are ill*,* weak*,* and older this can be challenging* (registered dietitian 2).

Three days of dietary recording per month as outlined in the draft protocol of the GAIN study was perceived as feasible by both healthcare professionals and patients (Process, CFIR construct Executing).

**Fig. 4:**
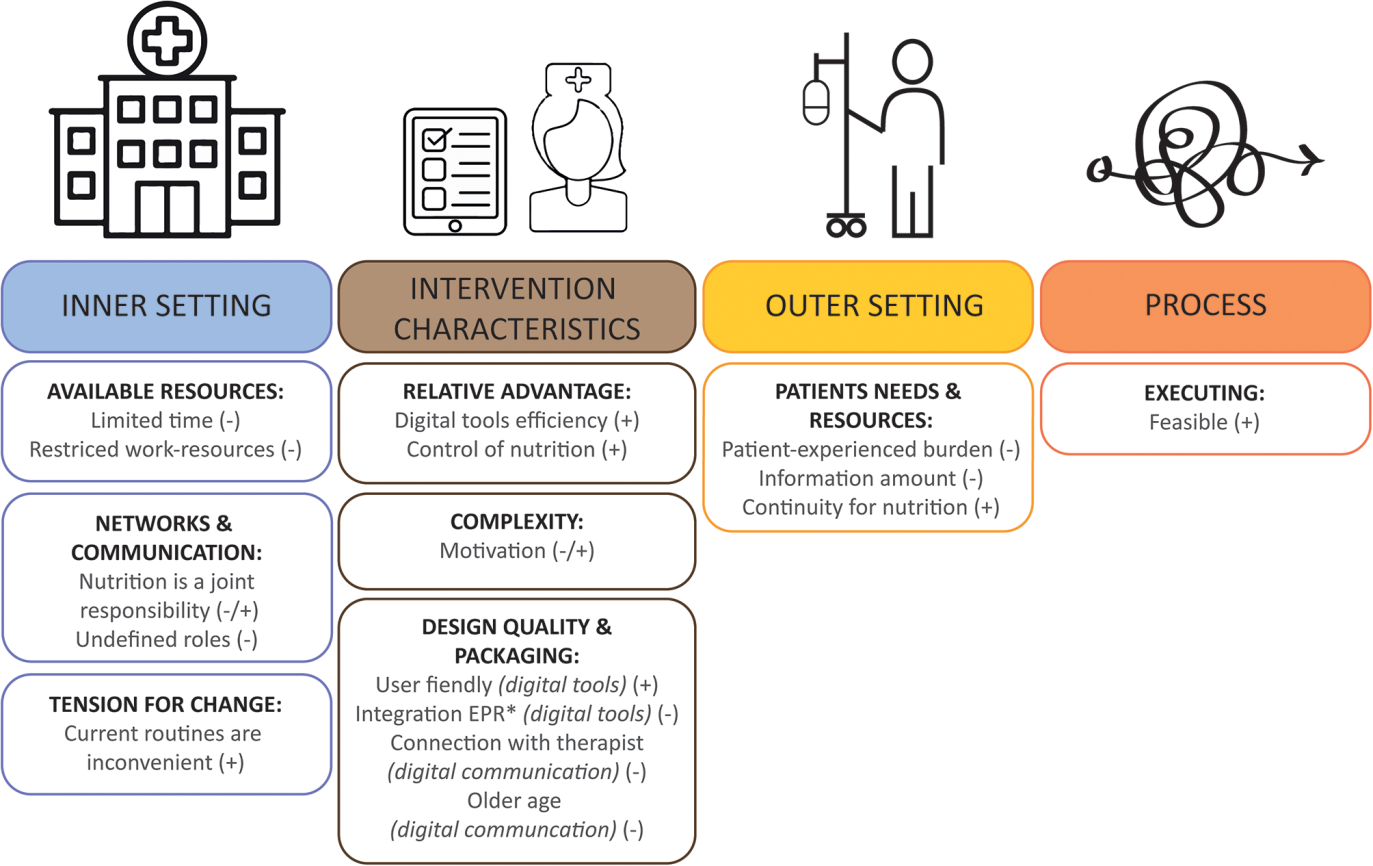
An overview of the main themes and belonging factors potentially influencing the implementation of the GAIN intervention. Barriers and facilitators are represented in the figure with – and +, respectively. *Electronic patient record

An overview of the results sorted into main themes and the affecting factors within these themes are given in Fig. 4.

## Discussion

This pre-implementation study explored healthcare professionals’ and patients’ views on the current nutritional support in the clinical cancer pathway and potential barriers and facilitators of the GAIN intervention including improved nutritional support for patients with cancer. This study found barriers related to the current nutritional support, such as limited resources and undefined roles concerning responsibility for providing nutritional support among healthcare professionals. Facilitators included a desire for change regarding current nutritional practice. The GAIN intervention was perceived as feasible and efficient with the use of a digital dietary tool. Contrary, potential barriers included a possible lack of technological skills among older patients, lack of motivation among patients in general and the potential added burden to the patients by participating in the intervention.

### Current practices of nutritional support

Exploring the current practices for providing nutritional support showed that most patients were screened for malnutrition by healthcare professionals assessing their weight and dietary intake. However, limited resources, including time and the number of healthcare professionals, as well as limited leadership engagement, were identified as barriers to delivering nutritional support. These findings are consistent with a Canadian review describing barriers and facilitators to implementing evidence-based guidelines for health outcomes in long-term care by McArthur et al. [22], who identified time constraints, inadequate staffing, and lack of organizational support as barriers. Similar findings have also previously been identified in Norwegian studies on the implementation of the MyFood tool in different contexts [28–30].

The healthcare respondents in the present study had different opinions on who was responsible for providing nutritional support but agreed that it was a joint responsibility. This finding highlights the importance of employing consistent and comprehensible language – using unified terms that patients are familiar with rather than advanced medical jargon dependent on occupation – when providing nutritional support to patients. Using a common language may assure that patients receive accurate information irrespective of the healthcare professional communicating the information. This was demonstrated in a systematic review which found that patients with cancer experienced confusion due to conflicting and unrelatable language, when receiving dietary advice from healthcare professionals [31].

### The improved nutritional support in the GAIN intervention

Both barriers and facilitators related to the GAIN intervention were identified. Old age and thereby potential lack of technological knowledge among the patients was seen as a potential barrier. Despite this expressed concern from healthcare professionals, none of the responding patients mentioned this as a barrier for them. Contrarily, it was mentioned that the majority of older adults nowadays are more comfortable with technology than perceived by the general public. These beliefs are supported in a study by Aure et al. [32] who found that older adults are both able and willing to use self-monitoring tools despite lack of prior experience with technology. Research also suggests that older adults are interested in providing data on diet and lifestyle, especially if it can help them improve their lifestyle [33, 34].

During the focus group discussion, the registered dietitians had different opinions on how frequently the patients in the intervention group in the GAIN study should record their dietary intake in MyFood between the study visits. Different suggestions were proposed, but it was agreed that 3 consecutive days, every month for 6 months, should be manageable for the patient and give an acceptable insight into the patient’s nutritional status for the healthcare professionals. The patients agreed that a 3-day dietary recording would be feasible. Although there are uncertainties connected to dietary records such as changing the usual dietary pattern for ease of recording or desirability to report food perceived as healthy, Kwan et al. [35] found that a 3-day recording was an acceptable approach for assessing the dietary intake in a similar study population recently diagnosed with cancer. The study also showed that error rates for completion and prevalence of missing data were lower if the patients received proper instructions on how to use the dietary registration tool [35]. This highlights the importance of technical support and training to facilitate the intervention and complies with what was argued by the respondents in the current study.

An important barrier to the implementation of improved nutritional support for patients with cancer was found when exploring the outer setting of the intervention. Several of the healthcare professionals included in this study expressed their concern regarding a potential patient-experienced burden. This burden was specific to the improved nutritional support, in terms of the extra follow-ups and communication with dietitians, in addition to the digital dietary registration at home combined with the cancer treatment. A load of psychosocial consequences, for instance emotions such as disappointment, guilt, and powerlessness were mentioned as possible consequences of the increased nutritional focus through dietary recording and following nutritional support. These emotions were also identified by Alberda et al. [36], who investigated the patient perspective of nutritional care and support in patients with head and neck or oesophageal cancer. The study found that some patients felt helpless when failing to meet their prescribed calorie goal, especially when their weight declined, and the next treatment intervention was a feeding tube [36]. Similarly, a qualitative study by Findlay et al. [37], aimed to understand the perspective of nutritional support among patients with head and neck cancer and their caregivers. They observed that patients felt overwhelmed and overloaded, having a multitude of questions as a consequence of a recent cancer diagnosis early in the cancer treatment pathway [37]. Furthermore, Findlay et al., found that the patients did not welcome the nutritional support before experiencing nutritional impact symptoms. However, the importance of such advice and treatment was shown to be highly appreciated in hindsight [37].

As opposed to the lack of control patients have over surgical approach and chemoradiation, eating is somewhat within their control. The desire to obtain information on diet and nutrition among patients with cancer seems to exceed the available scientifically based recommendations, especially if healthcare professionals do not convey this information to the patient [38]. A German cross-sectional study [39] found that the most common reason for change of diet or diet modification among patients with cancer were “to actively contribute something to the therapy” and “to support the therapy”. Less than 20% of the patients who changed or planned to change their diet in this study obtained information regarding nutrition from healthcare professionals. More than half of the patients, however, gathered the information on their own using diverse methods (e.g. the internet, friends, and family) [39].

An Italian Intersociety Working Group for Nutritional Support in Cancer Patients suggests that patients with head and neck, gastrointestinal, or lung cancer, in addition to patients with advanced disease stage or aggressive treatment such as high dose chemotherapy, radical radiotherapy, or combined chemoradiation should immediately be referred to a specialist in nutrition, i.e., registered dietitian, independent of malnutrition risk [40]. This corresponds to what was practiced in the study by Findlay et al. [37] and is comparable to the improved nutritional support that will be provided by the GAIN study. While the referral to a registered dietitian introduces an additional consideration for the patient during a troublesome time, receiving practical advice for managing common nutrition impact symptoms associated with their cancer diagnosis, stage or treatment can be beneficial. A literature review by Richards et al. [41], looked into the timing of nutritional intervention for patients with cancer. Twelve of the 15 included studies provided an early nutrition intervention to the intervention groups. Early nutrition interventions were found to improve health and nutrition outcomes [41]. Two studies compared the impact of early and late nutrition intervention, and both studies found that early intervention was more favorable than late intervention due to a significant reduction in weight loss, a significant improvement in treatment tolerance, and a significant decrease in unplanned hospitalizations [42, 43].

### Strengths and limitations

There are several limitations to this study. Amongst them are the limited number of participants from each group of respondents, which may restrict the comprehensiveness of perspectives captured within this population. Nevertheless, the collected data were rich and gave valuable insights into the key barriers and facilitators for nutritional support in cancer patients, and informed the planning of the implementation of the GAIN intervention into clinical practice. Despite the limited number of participants, both patient and healthcare professional perspectives were captured, providing valuable insights.

Another limitation is that none of the patients included in this study were diagnosed with the cancer diagnoses intended to be included in the GAIN study. Since this study found indications of an increased patient-experienced burden among the interviewed healthcare professionals it would be preferable to have an opinion from the patients with the actual diagnoses. However, the included patients received nutritional support simultaneously with cancer treatment, which is similar to the intervention in the GAIN study. Thus, one could expect the patients from these diagnostic groups to be comparable with the intended diagnostic groups in the intervention.

A strength of this study was the inclusion of several groups of healthcare professionals, which contributed to different perspectives on the existing and improved nutritional support.

Another strength was the use of an established framework in implementation science to identify potential barriers and facilitators of the current nutritional support and the upcoming GAIN intervention. While processing the data, an updated version of the CFIR was published [26]. Although there are several alterations in the structure of the updated version (i.e., relocations of domains and constructs), we do not believe that this would have affected the results in the present study as a comparison between the two versions of the framework is possible and feasible if necessary [19].

Lastly, emphasizing trustworthiness in the analysis, maintaining the transferability, dependability, and confirmability as described by Nowell et al. [44], was considered a strength.

## Conclusion

This study showed the perspectives of healthcare professionals and patients on the current nutritional support for patients with cancer and explored potential barriers and facilitators for implementing an improved nutritional support through the GAIN study. Barriers to providing nutritional support included limited resources and unclear roles among healthcare professionals. The desire for change in the nutrition practice was identified as a facilitator for improved nutrition support, as was the use of digital dietary tools among all respondents. However, potential barriers included older patients’ possible lack of technological skills, general patient motivation, and the increased patient-experienced burden. The identification of the potential barriers and facilitators will be used to plan the implementation of improved nutritional support for patients with cancer in the GAIN study. Our finding might also be relevant for similar research studies, planning to implement nutritional interventions using digital tools during the cancer treatment course.

### Electronic supplementary material

Below is the link to the electronic supplementary material.

Supplementary Material 1

Supplementary Material 2

Supplementary Material 3

Supplementary Material 4

## Data Availability

The datasets used and analyzed during the current study are available from the corresponding author upon reasonable request.

## Abbreviations

CFIR: The Consolidated Framework for Implementation Research
EPR: Electronic patient record
GAIN: Green Approach to Improved Nutritional support for patients with cancer
RCT: Randomized clinical trial
SIKT: Norwegian Agency for Shared Services in Education and Research
