# Supplementary material for: Barriers and facilitators of improved nutritional support for patients newly diagnosed with cancer: a pre-implementation study
Source: BMC Health Serv Res. 2024 Jul 15;24:815. doi: 10.1186/s12913-024-11288-2 (PMC11251100; doi:10.1186/s12913-024-11288-2)
Supplement: Supplementary file 1 — Supplementary Material 1 [file 12913_2024_11288_MOESM1_ESM.docx]

INTERVIEW GUIDE PATIENTS

| Introduction | Information about the project, purpose of the interview, practical information (audio recording and confidentiality), brief introduction of the participant (cancer diagnosis, age and gender), questions from the participant |
| --- | --- |
| About nutrition | How do you experience today's offer in terms of nutritional follow-up?   - In the hospitals? - Here at the outpatient clinic?   What does it take for you to feel well taken care of in terms of nutrition?  Follow-up:   - How? - What do you think about digital versus telephone follow-up?   - What is good and less good about digital follow-up?   - What is good and less good about telephonically? |
| Demonstration of MyFood | Show screenshots/demonstration from application and web solution.   1. Registration of patient 2. Record nutrition 3. Evaluation of intake compared to needs |
| Use of MyFood | How can such a tool be used by you?  What kind of potential does the use of MyFood have?   - For patients? - For therapists?   - For nurses, doctors or others? - To what extent can MyFood contribute to better follow-up? - To what extent does the tool correspond with perceptions of good nutritional follow-up?   Challenges with use?   - What does it take to use MyFood? - Complexity? |
| Summary and conclusion | Summarize main points   - About the use of an electronic tool in clinical practice |
