# Supplementary material for: Barriers and facilitators of improved nutritional support for patients newly diagnosed with cancer: a pre-implementation study
Source: BMC Health Serv Res. 2024 Jul 15;24:815. doi: 10.1186/s12913-024-11288-2 (PMC11251100; doi:10.1186/s12913-024-11288-2)
Supplement: Supplementary file 2 — Supplementary Material 2 [file 12913_2024_11288_MOESM2_ESM.docx]

INTERVIEW GUIDE FOCUS GROUP DIETITIANS

| Introduction | Information about the project, purpose of the interview, practical information (audio recording and confidentiality), brief introduction of participants (name tag), questions from participants |
| --- | --- |
| About the work with nutrition | How is the nutrition outpatient clinic organized today? (Referrals, regular patient groups?)  How do you work with nutrition and the patients at the outpatient clinic?   - Risk assessment/screening?   - How?   - Which patients? - Routines:   - How is patients’ intake and need established?   - Follow-up of patients     - Which patients are followed up?     - How are they followed up? (physically at the clinic or from home via video or telephone consultations)     - What is good and what is challenging about these types of follow-ups? (please say something about the difference between physical vs digital, and digital vs telephone) - The patients' experience of the nutritional follow-up, what impressions are you left with? - What are you satisfied with in today's practice?   - What could be better? - Challenges? Why? Examples? - The role of nutrition in illness, treatment and recovery?   - How are the patients doing at home? |
| Demonstration of MyFood | Presentation of screenshots/demonstration from app and web solution.   1. Registration of patient 2. Recording nutritional intake 3. Evaluation of intake compared to needs 4. Report and feedback |
| Use of MyFood | How can MyFood be used in practice at an outpatient clinic like this? What is the potential of the application?   - For patients? - For employees?   - For partners outside the clinic such as nurses, health care providers, doctors or others? - To what extent can MyFood contribute to better follow-up of patients? - To what extent does the tool correspond with perceptions of good nutritional follow-up? - Thoughts or suggestions for the nutritional advice that is automatically generated? What is good, what is less good? What value does it have?   Challenges with use?   - What does it take to use MyFood? (Integration EPR?) - Complexity? |
| Design | What do you think about the layout, design, content and layout?   - What is good? - Something missing? |
| Intervention study | - Thoughts on conducting the study at the Center for Clinical Nutrition? - What do you think about close follow-up of the relevant patient groups (gynecological and bowel cancer)? - Self-registration for 3 consecutive days, once every month between visits. What do you think? - What do you need from the study (GAIN/me)? - What kind of training do you need to use MyFood? - Specific key people? - Considerations to decide on? - How can we (I) in the project group contribute to the implementation going as well as possible? |
| Summary and conclusion | Summarize main points - what has emerged along the way.   - Anything else to add?   - About the nutrition work at the clinic   - About the use of an electronic tool in clinical practice   - About the implementation of the intervention study on the ward |
