# Supplementary material for: Barriers and facilitators of improved nutritional support for patients newly diagnosed with cancer: a pre-implementation study
Source: BMC Health Serv Res. 2024 Jul 15;24:815. doi: 10.1186/s12913-024-11288-2 (PMC11251100; doi:10.1186/s12913-024-11288-2)
Supplement: Supplementary file 3 — Supplementary Material 3 [file 12913_2024_11288_MOESM3_ESM.docx]

INTERVIEW GUIDE NURSE/PHYSISIAN

| Introduction | Information about the project, purpose of the interview, practical information (audio recording and confidentiality), brief introduction of the participant, questions from the participant |
| --- | --- |
| Work experience and organization | How long have you worked at in this hospital?   - Elsewhere? (clinical)   Do you cover more departments than this one? |
| About nutritional support | How is the nutrition support for patients organized on this ward?   - Risk assessment/screening?   - How?   - Who is responsible?   - Which patients are assessed? - Routines:   - Mapping the patient's intake and needs:     - How is it done as of today?     - If there are lists, what is done with them?     - Specific examples?   - Follow-up of patients     - Which patients are followed up?     - How are they followed up? (physically at the clinic or from home via video or telephone consultations)     - What is good and challenging about this type of follow-up? - Responsibilities:   - Who has the main responsibility for the nutritional support?   - Division of responsibility – clear?   - Are physicians responsible? Do they take responsibility? - Management commitment:   - Signals from the management that nutritional work should be prioritized?   - What is being done to support the nutritional work? - The patients' experience of the nutritional follow-up   - Food, meals and nutrition on the ward? - What are you satisfied with in today's practice? What could be better? - Challenges? - The role of nutrition in illness, treatment and recovery in those patients you treat as of today? |
| Demonstration of MyFood | Show screenshots/demonstration from application and web solution.   1. Registration of patient 2. Record nutrition 3. Evaluation of intake compared to needs 4. Report and feedback |
| Use of MyFood | How can MyFood be used in practice?  What kind of potential is the use of MyFood in the department?   - For patients? - For employees?   - For nurses, physicians or others? - To what extent can MyFood contribute to better follow-up of patients? - To what extent does the tool correspond with perceptions of good nutritional follow-up? - Challenges with use? Complexity? - What does it take to use MyFood? |
| Design | What do you think about the layout, design, content and layout?   - What is good? - Something missing? |
| Intervention study | - Thoughts on carrying out the study? - What do you think about closer nutritional follow-up of the relevant patient groups (patients with cervical and lower GI cancer)? - What kind of training do you need to use MyFood? - Specific key people? - Are there conditions in the department that are important to take into account? - How can we (I) in the project group contribute to the implementation going as well as possible? |
| Summary and conclusion | Summarize main points   - Anything else to add?   - About the nutrition work at the clinic   - About the use of an electronic tool in clinical practice   - About the implementation of the intervention study on the ward |
