# Supplementary material for: Barriers and facilitators of improved nutritional support for patients newly diagnosed with cancer: a pre-implementation study
Source: BMC Health Serv Res. 2024 Jul 15;24:815. doi: 10.1186/s12913-024-11288-2 (PMC11251100; doi:10.1186/s12913-024-11288-2)
Supplement: Supplementary file 4 — Supplementary Material 4 [file 12913_2024_11288_MOESM4_ESM.docx]

**Focus group discussion, registered dietitians**

Questions about background

1. Gender: Female Male

2. Age:_________ years

3. Education: __________________________________

Education finished:________ year

Focus area(s) (allergies, intestinal problems, patients with cancer etc):

1.________________________________

 2.________________________________

3.________________________________

4. Work experience at the outpatient clinic:__________ months or years (specify)

5. Work experience as a registered dietitian at other departments/hospitals:__________
